# (*E*)-2-[(2-Ethyl­phen­yl)imino­meth­yl]-6-methoxy­phenol

**DOI:** 10.1107/S1600536809055573

**Published:** 2010-01-09

**Authors:** Serap Yazıcı, Çiğdem Albayrak, İsmail Gümrükçüoğlu, İsmet Şenel, Orhan Büyükgüngör

**Affiliations:** a Department of Physics, Faculty of Arts and Sciences, Ondokuz Mayıs University, TR-55139 Kurupelit–Samsun, Turkey; b Sinop Faculty of Education, Sinop University, TR-57000 Sinop, Turkey; c Department of Chemistry, Ondokuz Mayıs University, TR-55139 Kurupelit–Samsun, Turkey

## Abstract

The mol­ecule of the title compound, C_16_H_17_NO_2_, adopts the phenol–imine tautomeric form with a strong intra­molecular O—H⋯N hydrogen bond and an *E* conformation with respect to the azomethine C=N bond. The dihedral angle between the aromatic rings is 21.23 (9)°. The ethyl group is disordered over two orientations with occupancies of 0.598 (6) and 0.402 (6). In the crystal, the mol­ecules are linked into chains along the *b* axis by C—H⋯π inter­actions.

## Related literature

For general background to *o*-hydr­oxy Schiff bases, see: Stewart & Lingafelter (1959 ▶); Calligaris *et al.* (1972 ▶); Maslen & Waters (1975 ▶). For the photochromic and thermochromic characteristics of Schiff base compounds, see: Cohen *et al.* (1964 ▶); Moustakali-Mavridis *et al.* (1980 ▶); Hadjoudis *et al.* (1987 ▶); Xu *et al.* (1994 ▶). For a related structure, see: Yüce *et al.* (2004 ▶).
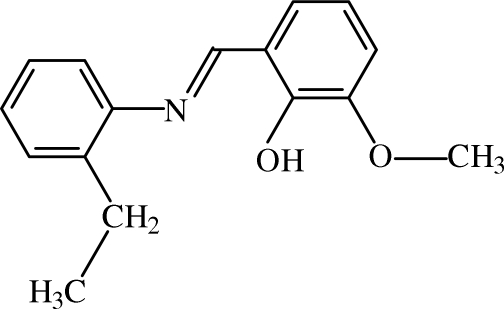


## Experimental

### 

#### Crystal data

C_16_H_17_NO_2_
*M* *_r_* = 255.31
Monoclinic, 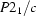

*a* = 18.2379 (7) Å
*b* = 5.2044 (2) Å
*c* = 15.0950 (7) Å
β = 113.788 (3)°
*V* = 1311.05 (9) Å^3^
*Z* = 4
Mo *K*α radiation
μ = 0.09 mm^−1^
*T* = 150 K
0.58 × 0.39 × 0.08 mm

#### Data collection

Stoe IPDS II diffractometer
Absorption correction: integration (*X-RED32*; Stoe & Cie, 2002 ▶) *T*_min_ = 0.961, *T*_max_ = 0.993
18335 measured reflections
3014 independent reflections
2353 reflections with *I* > 2σ(*I*)
*R*_int_ = 0.072

#### Refinement

*R*[*F* ^2^ > 2σ(*F* ^2^)] = 0.044
*wR*(*F* ^2^) = 0.115
*S* = 1.03
3014 reflections
196 parameters
H atoms treated by a mixture of independent and constrained refinement
Δρ_max_ = 0.26 e Å^−3^
Δρ_min_ = −0.32 e Å^−3^

### 

Data collection: *X-AREA* (Stoe & Cie, 2002 ▶); cell refinement: *X-AREA*; data reduction: *X-RED32* (Stoe & Cie, 2002 ▶); program(s) used to solve structure: *SHELXS97* (Sheldrick, 2008 ▶); program(s) used to refine structure: *SHELXL97* (Sheldrick, 2008 ▶); molecular graphics: *ORTEP-3 for Windows* (Farrugia, 1997 ▶); software used to prepare material for publication: *WinGX* (Farrugia, 1999 ▶).

## Supplementary Material

Crystal structure: contains datablocks I, global. DOI: 10.1107/S1600536809055573/ci5005sup1.cif

Structure factors: contains datablocks I. DOI: 10.1107/S1600536809055573/ci5005Isup2.hkl

Additional supplementary materials:  crystallographic information; 3D view; checkCIF report

## Figures and Tables

**Table 1:** Hydrogen-bond geometry (Å, °) *Cg*1 is the centroid of the C8–C13 ring.

*D*—H⋯*A*	*D*—H	H⋯*A*	*D*⋯*A*	*D*—H⋯*A*
O1—H1⋯N1	0.98 (2)	1.68 (2)	2.6023 (15)	156 (2)
C14—H14c⋯*Cg*1^i^	0.96	2.83	3.6241 (18)	141

Symmetry code: (i) 
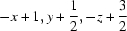
.

**Fig. 1.**
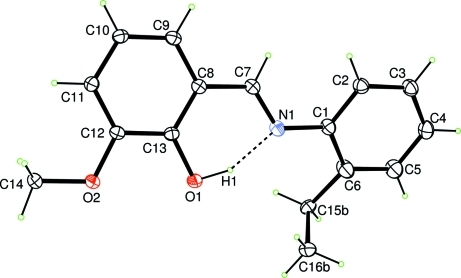
The molecular structure of the title compound, with the atomic numbering scheme. Displacement ellipsoids are drawn at the 30% probability level. The dashed line indicates a hydrogen bond. Only the major disorder component is shown.

**Fig. 2.**
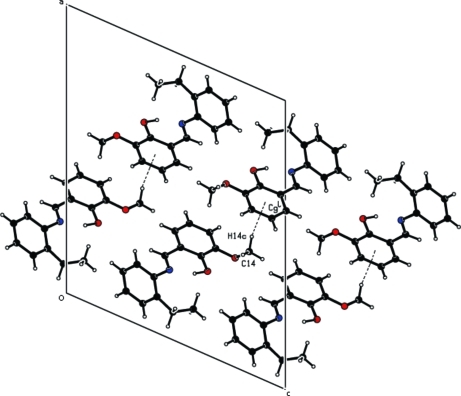
A partial packing diagram of the title compound. Dashed lines indicate C—H···π interactions.

## supplementary crystallographic information

### Comment

*o*-Hydroxy Schiff bases derived from the reaction of *o*-hydroxyaldehydes with aniline have been examined extensively (Steward & Lingafelter, 1959; Calligaris *et al.*, 1972; Maslen & Waters, 1975). Schiff base compounds display interesting photochromic and thermochromic features and can be classified in terms of these (Cohen *et al.*, 1964; Moustakali-Mavridis *et al.*, 1980; Hadjoudis *et al.*, 1987). Photo- and thermochromism arise *via* H atom transfer from the hydroxy O atom to the N atom (Hadjoudis *et al.*, 1987; Xu *et al.*, 1994).

The molecule of the title compound (Fig. 1) exists in the phenol-imine form which is confirmed by C13—O1 and C7—N1 bond distances. These distances agree with the corresponding distances in 1-{4-[(2-hydroxybenzylidene)amino]phenyl}ethanone, a related structure [C—O = 1.3500 (17) and C—N = 1.2772 (16) Å; Yüce *et al.*, 2004].

The title molecule is not planar; the dihedral angle between the two benzene rings is 21.23 (9)°. An intramolecular O1—H1···N1 hydrogen bond (Fig. 1) generates an S(6) ring-motif.

The crystal structure is stabilized by weak C—H···π interactions involving H14C and C8–C13 ring (Fig. 2).

### Experimental

A solution of 3-methoxysalicylaldehyde (0.5 g 3.3 mmol) in ethanol (20 ml) was added to a solution of 2-ethylaniline (0.4 g 3.3 mmol) in  ethanol (20 ml). The reaction mixture was stirred for 1 h under reflux. Single crystals of the title compound were obtained by slow evaporation of an ethanol solution (yield 72%, m.p. 339-340 K).

### Refinement

The ethyl group is disordered over two orientations with occupancies of 0.598 (6) and 0.402 (6). Atom H1 was located in a difference map and refined freely. The remaining H atoms were placed in calculated positions and constrained to ride on their parents atoms, with C-H = 0.93–0.96 Å and *U*_iso_(H) = 1.2*U*_eq_(C) and 1.5*U*_eq_(C_methyl_).

### Crystal data

**Table:**

C_16_H_17_NO_2_	*F*(000) = 544
*M**_r_* = 255.31	*D*_x_ = 1.293 Mg m^−^^3^
Monoclinic, *P*2_1_/*c*	Mo *K*α radiation, λ = 0.71073 Å
Hall symbol: -P 2ybc	Cell parameters from 2353 reflections
*a* = 18.2379 (7) Å	θ = 1.5–28.0°
*b* = 5.2044 (2) Å	µ = 0.09 mm^−^^1^
*c* = 15.0950 (7) Å	*T* = 150 K
β = 113.788 (3)°	Plate, brown
*V* = 1311.05 (9) Å^3^	0.58 × 0.39 × 0.08 mm
*Z* = 4	

### Data collection

**Table:**

Stoe IPDS II diffractometer	3014 independent reflections
Radiation source: fine-focus sealed tube	2353 reflections with *I* > 2σ(*I*)
graphite	*R*_int_ = 0.072
Detector resolution: 6.67 pixels mm^-1^	θ_max_ = 27.6°, θ_min_ = 2.4°
ω scan	*h* = −23→23
Absorption correction: integration (*X-RED32*; Stoe & Cie, 2002)	*k* = −6→6
*T*_min_ = 0.961, *T*_max_ = 0.993	*l* = −19→19
18335 measured reflections	

### Refinement

**Table:**

Refinement on *F*^2^	Secondary atom site location: difference Fourier map
Least-squares matrix: full	Hydrogen site location: inferred from neighbouring sites
*R*[*F*^2^ > 2σ(*F*^2^)] = 0.044	H atoms treated by a mixture of independent and constrained refinement
*wR*(*F*^2^) = 0.115	*w* = 1/[σ^2^(*F*_o_^2^) + (0.052*P*)^2^ + 0.279*P*] where *P* = (*F*_o_^2^ + 2*F*_c_^2^)/3
*S* = 1.03	(Δ/σ)_max_ = 0.001
3014 reflections	Δρ_max_ = 0.26 e Å^−^^3^
196 parameters	Δρ_min_ = −0.32 e Å^−^^3^
0 restraints	Extinction correction: *SHELXL97* (Sheldrick, 2008), Fc^*^=kFc[1+0.001xFc^2^λ^3^/sin(2θ)]^-1/4^
Primary atom site location: structure-invariant direct methods	Extinction coefficient: 0.024 (3)

### Special details

**Table:**

Experimental. 320 frames, detector distance = 100 mm
Geometry. All e.s.d.'s (except the e.s.d. in the dihedral angle between two l.s. planes) are estimated using the full covariance matrix. The cell e.s.d.'s are taken into account individually in the estimation of e.s.d.'s in distances, angles and torsion angles; correlations between e.s.d.'s in cell parameters are only used when they are defined by crystal symmetry. An approximate (isotropic) treatment of cell e.s.d.'s is used for estimating e.s.d.'s involving l.s. planes.
Refinement. Refinement of *F*^2^ against ALL reflections. The weighted *R*-factor *wR* and goodness of fit *S* are based on *F*^2^, conventional *R*-factors *R* are based on *F*, with *F* set to zero for negative *F*^2^. The threshold expression of *F*^2^ > σ(*F*^2^) is used only for calculating *R*-factors(gt) *etc*. and is not relevant to the choice of reflections for refinement. *R*-factors based on *F*^2^ are statistically about twice as large as those based on *F*, and *R*- factors based on ALL data will be even larger.

### Fractional atomic coordinates and isotropic or equivalent isotropic displacement parameters (Å^2^)

**Table:**

	*x*	*y*	*z*	*U*_iso_*/*U*_eq_	Occ. (<1)
C16A	0.0701 (3)	0.3212 (10)	0.5111 (3)	0.0532 (15)	0.402 (6)
H16A	0.0628	0.3425	0.5702	0.080*	0.402 (6)
H16B	0.0188	0.3027	0.4581	0.080*	0.402 (6)
H16C	0.0970	0.4691	0.5006	0.080*	0.402 (6)
C15A	0.1206 (3)	0.0813 (10)	0.5183 (4)	0.0411 (12)	0.402 (6)
H15A	0.0944	−0.0727	0.5274	0.049*	0.402 (6)
H15B	0.1734	0.0953	0.5705	0.049*	0.402 (6)
C15B	0.09657 (17)	0.2040 (7)	0.4866 (2)	0.0380 (9)	0.598 (6)
H15C	0.1114	0.3839	0.4967	0.046*	0.598 (6)
H15D	0.0388	0.1903	0.4639	0.046*	0.598 (6)
C16B	0.13777 (19)	0.0531 (6)	0.5796 (3)	0.0470 (9)	0.598 (6)
H16D	0.1220	0.1202	0.6285	0.070*	0.598 (6)
H16E	0.1948	0.0681	0.6009	0.070*	0.598 (6)
H16F	0.1226	−0.1244	0.5682	0.070*	0.598 (6)
H1	0.2630 (14)	0.462 (4)	0.5862 (17)	0.077 (7)*	
O1	0.29046 (6)	0.5645 (2)	0.64484 (7)	0.0387 (3)	
N1	0.23740 (7)	0.3745 (3)	0.47070 (8)	0.0376 (3)	
C12	0.38182 (8)	0.9118 (3)	0.68144 (9)	0.0314 (3)	
O2	0.37858 (6)	0.9107 (2)	0.77070 (7)	0.0386 (3)	
C9	0.38032 (8)	0.8881 (3)	0.49637 (10)	0.0344 (3)	
H9	0.3798	0.8812	0.4345	0.041*	
C13	0.33418 (7)	0.7266 (3)	0.61589 (9)	0.0306 (3)	
C8	0.33213 (7)	0.7187 (3)	0.52183 (9)	0.0315 (3)	
C14	0.42206 (9)	1.1110 (3)	0.83509 (10)	0.0406 (3)	
H14A	0.4162	1.0939	0.8952	0.061*	
H14B	0.4015	1.2747	0.8066	0.061*	
H14C	0.4777	1.0989	0.8468	0.061*	
C11	0.42846 (8)	1.0776 (3)	0.65443 (10)	0.0338 (3)	
H11	0.4602	1.1993	0.6982	0.041*	
C7	0.27988 (8)	0.5403 (3)	0.45018 (10)	0.0346 (3)	
H7	0.2772	0.5468	0.3874	0.042*	
C2	0.20659 (9)	0.1179 (3)	0.32266 (10)	0.0407 (3)	
H2	0.2493	0.1925	0.3133	0.049*	
C10	0.42817 (8)	1.0634 (3)	0.56190 (10)	0.0347 (3)	
H10	0.4605	1.1732	0.5447	0.042*	
C3	0.16088 (10)	−0.0684 (3)	0.25904 (11)	0.0474 (4)	
H3	0.1729	−0.1186	0.2073	0.057*	
C1	0.18945 (8)	0.1950 (3)	0.40048 (10)	0.0378 (3)	
C4	0.09745 (10)	−0.1796 (3)	0.27231 (11)	0.0479 (4)	
H4	0.0666	−0.3053	0.2298	0.057*	
C6	0.12496 (10)	0.0848 (4)	0.41384 (13)	0.0594 (5)	
C5	0.08014 (10)	−0.1034 (4)	0.34882 (13)	0.0568 (5)	
H5	0.0373	−0.1794	0.3575	0.068*	

### Atomic displacement parameters (Å^2^)

**Table:**

	*U*^11^	*U*^22^	*U*^33^	*U*^12^	*U*^13^	*U*^23^
C16A	0.049 (2)	0.056 (3)	0.061 (3)	0.001 (2)	0.028 (2)	−0.013 (2)
C15A	0.041 (2)	0.041 (3)	0.043 (4)	−0.007 (2)	0.019 (2)	0.004 (2)
C15B	0.0310 (13)	0.0355 (19)	0.0482 (16)	0.0008 (13)	0.0168 (12)	0.0063 (14)
C16B	0.0583 (17)	0.0449 (16)	0.042 (2)	−0.0002 (13)	0.0248 (15)	0.0062 (13)
O1	0.0388 (5)	0.0449 (6)	0.0382 (5)	−0.0100 (5)	0.0216 (4)	−0.0055 (5)
N1	0.0279 (5)	0.0472 (7)	0.0369 (6)	−0.0015 (5)	0.0124 (5)	−0.0066 (5)
C12	0.0324 (6)	0.0346 (7)	0.0296 (6)	0.0033 (6)	0.0151 (5)	0.0015 (5)
O2	0.0447 (5)	0.0440 (6)	0.0327 (5)	−0.0101 (5)	0.0214 (4)	−0.0068 (4)
C9	0.0384 (7)	0.0358 (7)	0.0318 (6)	0.0057 (6)	0.0169 (6)	0.0043 (5)
C13	0.0271 (6)	0.0332 (7)	0.0338 (6)	0.0025 (5)	0.0146 (5)	0.0020 (5)
C8	0.0290 (6)	0.0337 (7)	0.0315 (6)	0.0053 (5)	0.0117 (5)	0.0016 (5)
C14	0.0491 (8)	0.0413 (9)	0.0362 (7)	−0.0070 (7)	0.0220 (6)	−0.0083 (6)
C11	0.0358 (7)	0.0316 (7)	0.0345 (7)	−0.0002 (6)	0.0148 (6)	0.0000 (6)
C7	0.0307 (6)	0.0404 (8)	0.0318 (6)	0.0055 (6)	0.0116 (5)	−0.0002 (6)
C2	0.0402 (7)	0.0450 (9)	0.0350 (7)	−0.0007 (6)	0.0132 (6)	−0.0004 (6)
C10	0.0381 (7)	0.0329 (7)	0.0372 (7)	0.0013 (6)	0.0193 (6)	0.0050 (6)
C3	0.0531 (9)	0.0507 (10)	0.0350 (7)	−0.0020 (8)	0.0143 (7)	−0.0054 (7)
C1	0.0299 (6)	0.0454 (9)	0.0340 (7)	−0.0001 (6)	0.0088 (5)	−0.0047 (6)
C4	0.0426 (8)	0.0488 (9)	0.0396 (8)	−0.0027 (7)	0.0035 (6)	−0.0058 (7)
C6	0.0407 (8)	0.0860 (14)	0.0566 (10)	−0.0213 (9)	0.0248 (8)	−0.0273 (10)
C5	0.0376 (8)	0.0732 (13)	0.0584 (10)	−0.0174 (8)	0.0180 (8)	−0.0171 (9)

### Geometric parameters (Å, °)

**Table:**

C16A—C15A	1.529 (7)	C9—C10	1.370 (2)
C16A—H16A	0.96	C9—C8	1.4035 (19)
C16A—H16B	0.96	C9—H9	0.93
C16A—H16C	0.96	C13—C8	1.4058 (17)
C15A—C6	1.611 (5)	C8—C7	1.4521 (19)
C15A—H15A	0.97	C14—H14A	0.96
C15A—H15B	0.97	C14—H14B	0.96
C15B—C16B	1.516 (5)	C14—H14C	0.96
C15B—C6	1.523 (3)	C11—C10	1.3966 (18)
C15B—H15C	0.97	C11—H11	0.93
C15B—H15D	0.97	C7—H7	0.93
C16B—H16D	0.96	C2—C3	1.382 (2)
C16B—H16E	0.96	C2—C1	1.391 (2)
C16B—H16F	0.96	C2—H2	0.93
O1—C13	1.3490 (16)	C10—H10	0.93
O1—H1	0.98 (2)	C3—C4	1.378 (2)
N1—C7	1.2781 (19)	C3—H3	0.93
N1—C1	1.4193 (18)	C1—C6	1.394 (2)
C12—O2	1.3722 (15)	C4—C5	1.373 (2)
C12—C11	1.3834 (19)	C4—H4	0.93
C12—C13	1.4034 (19)	C6—C5	1.394 (2)
O2—C14	1.4274 (17)	C5—H5	0.93
			
C15A—C16A—H16A	109.5	C9—C8—C7	119.54 (12)
C15A—C16A—H16B	109.5	C13—C8—C7	120.87 (12)
H16A—C16A—H16B	109.5	O2—C14—H14A	109.5
C15A—C16A—H16C	109.5	O2—C14—H14B	109.5
H16A—C16A—H16C	109.5	H14A—C14—H14B	109.5
H16B—C16A—H16C	109.5	O2—C14—H14C	109.5
C16A—C15A—C6	100.8 (4)	H14A—C14—H14C	109.5
C16A—C15A—H15A	111.6	H14B—C14—H14C	109.5
C6—C15A—H15A	111.6	C12—C11—C10	120.47 (13)
C16A—C15A—H15B	111.6	C12—C11—H11	119.8
C6—C15A—H15B	111.6	C10—C11—H11	119.8
H15A—C15A—H15B	109.4	N1—C7—C8	122.06 (12)
C16B—C15B—C6	105.7 (3)	N1—C7—H7	119.0
C16B—C15B—H15C	110.6	C8—C7—H7	119.0
C6—C15B—H15C	110.6	C3—C2—C1	120.71 (14)
C16B—C15B—H15D	110.6	C3—C2—H2	119.6
C6—C15B—H15D	110.6	C1—C2—H2	119.6
H15C—C15B—H15D	108.7	C9—C10—C11	120.11 (13)
C15B—C16B—H16D	109.5	C9—C10—H10	119.9
C15B—C16B—H16E	109.5	C11—C10—H10	119.9
H16D—C16B—H16E	109.5	C4—C3—C2	120.00 (14)
C15B—C16B—H16F	109.5	C4—C3—H3	120.0
H16D—C16B—H16F	109.5	C2—C3—H3	120.0
H16E—C16B—H16F	109.5	C2—C1—C6	119.58 (14)
C13—O1—H1	101.6 (13)	C2—C1—N1	122.67 (13)
C7—N1—C1	120.97 (12)	C6—C1—N1	117.68 (13)
O2—C12—C11	124.55 (12)	C5—C4—C3	119.48 (15)
O2—C12—C13	115.46 (11)	C5—C4—H4	120.3
C11—C12—C13	119.99 (12)	C3—C4—H4	120.3
C12—O2—C14	115.69 (10)	C5—C6—C1	118.45 (15)
C10—C9—C8	120.50 (12)	C5—C6—C15B	121.37 (16)
C10—C9—H9	119.7	C1—C6—C15B	119.27 (17)
C8—C9—H9	119.7	C5—C6—C15A	115.8 (2)
O1—C13—C12	118.68 (11)	C1—C6—C15A	121.60 (19)
O1—C13—C8	122.03 (12)	C4—C5—C6	121.78 (16)
C12—C13—C8	119.27 (12)	C4—C5—H5	119.1
C9—C8—C13	119.58 (12)	C6—C5—H5	119.1
			
C11—C12—O2—C14	5.35 (19)	C3—C2—C1—N1	176.31 (14)
C13—C12—O2—C14	−175.41 (12)	C7—N1—C1—C2	25.3 (2)
O2—C12—C13—O1	−0.49 (18)	C7—N1—C1—C6	−157.73 (16)
C11—C12—C13—O1	178.79 (12)	C2—C3—C4—C5	0.2 (3)
O2—C12—C13—C8	178.24 (11)	C2—C1—C6—C5	0.9 (3)
C11—C12—C13—C8	−2.48 (19)	N1—C1—C6—C5	−176.19 (17)
C10—C9—C8—C13	−1.1 (2)	C2—C1—C6—C15B	−168.4 (2)
C10—C9—C8—C7	177.95 (13)	N1—C1—C6—C15B	14.5 (3)
O1—C13—C8—C9	−178.51 (12)	C2—C1—C6—C15A	156.9 (3)
C12—C13—C8—C9	2.80 (19)	N1—C1—C6—C15A	−20.1 (4)
O1—C13—C8—C7	2.45 (19)	C16B—C15B—C6—C5	95.4 (3)
C12—C13—C8—C7	−176.23 (12)	C16B—C15B—C6—C1	−95.6 (3)
O2—C12—C11—C10	179.66 (13)	C16B—C15B—C6—C15A	7.7 (3)
C13—C12—C11—C10	0.4 (2)	C16A—C15A—C6—C5	−108.3 (3)
C1—N1—C7—C8	−177.25 (12)	C16A—C15A—C6—C1	95.1 (3)
C9—C8—C7—N1	176.91 (13)	C16A—C15A—C6—C15B	0.4 (3)
C13—C8—C7—N1	−4.1 (2)	C3—C4—C5—C6	0.1 (3)
C8—C9—C10—C11	−1.0 (2)	C1—C6—C5—C4	−0.7 (3)
C12—C11—C10—C9	1.3 (2)	C15B—C6—C5—C4	168.4 (2)
C1—C2—C3—C4	0.1 (3)	C15A—C6—C5—C4	−158.1 (3)
C3—C2—C1—C6	−0.6 (3)		

### Hydrogen-bond geometry (Å, °)

**Table:**

Cg1 is the centroid of the C8–C13 ring.

**Table:**

*D*—H···*A*	*D*—H	H···*A*	*D*···*A*	*D*—H···*A*
O1—H1···N1	0.98 (2)	1.68 (2)	2.6023 (15)	156 (2)
C14—H14c···Cg1^i^	0.96	2.83	3.6241 (18)	141

Symmetry codes: (i) −*x*+1, *y*+1/2, −*z*+3/2.
